# Irradiation-induced nasopharyngeal necrosis (INN) in newly diagnosed nasopharyngeal carcinoma treated by intensity-modulated radiation therapy: clinical characteristics and the influence of treatment strategies

**DOI:** 10.1186/s13014-022-01980-0

**Published:** 2022-01-21

**Authors:** Yi Xu, Yang Liu, Zekun Wang, Jingbo Wang, Jianghu Zhang, Xuesong Chen, Runye Wu, Qingfeng Liu, Yuan Qu, Kai Wang, Xiaodong Huang, Jingwei Luo, Li Gao, Guozhen Xu, Ye Zhang, Junlin Yi

**Affiliations:** Department of Radiation Oncology, National Cancer Center/National Clinical Research Center for Cancer/Cancer Hospital, Chinese Academy of Medical Sciences (CAMS) and Peking Union Medical College (PUMC), No17, Panjiayuan Nanli, Chaoyang District, Beijing, 100021 People’s Republic of China

**Keywords:** Irradiation-induced nasopharyngeal necrosis, Primary nasopharyngeal carcinoma, Intensity-modulated radiation therapy

## Abstract

**Purpose:**

To define the clinical characteristics of irradiation-induced nasopharyngeal necrosis (INN) after intensity-modulated radiotherapy (IMRT) and identify the influence of treatment strategies on INN in primary nasopharyngeal carcinoma (NPC) patients.

**Patients and methods:**

From 2008 to 2019, NPC patients pathologically diagnosed with INN after primary IMRT were reviewed. Those patients were matched with propensity scores for patients without INN in our center. The impact of treatment strategies on INN occurrence was assessed using univariate and multivariate logistic regression analysis.

**Results:**

The incidence rate of INN was 1.9% among the primary NPC population, and 53 patients with INN were enrolled. Headache and foul odor were the main symptoms, and 71.7% of cases had pseudomembrane during or at the end of radiotherapy. All patients were in early or middle stage INN, and no one presented with skull-based osteoradionecrosis. Then 212 non-INN patients were included based on propensity scores match. Overall survival (*p* = 0.248) and progression-free survival (*p* = 0.266) curves were similar between the INN and non-INN groups. Treatment strategies including combining chemotherapy or molecular targeted therapy with radiotherapy were not associated with INN occurrence, while boost dose (OR 7.360; 95% CI 2.301–23.547; *p* = 0.001) was a predictor factor for it. However, the optimal threshold for an accumulated dose to predict INN's occurrence was failed to determine.

**Conclusion:**

In the IMRT era, the severity of INN in primary NPC patients is lessened. This study showed that treatment strategies contributed little to develop INN, while the accumulated dose of radiation may relate to its occurrence.

## Introduction

With the development of radiotherapy techniques, intensity-modulated radiation therapy (IMRT), volumetric modulated arc therapy (VMAT), and helical tomotherapy (TOMO) have been wildly used for nasopharyngeal carcinoma (NPC) treatment in recent years [1–4]. Compared to 3D-conformal radiotherapy, intensity-modulated techniques improve local–regional control and overall survival by delivering high doses to the tumor while decreasing the dose to the nearby normal tissue [5]. Therefore, high dose irradiation-induced nasopharyngeal necrosis (INN) as a severe adverse complication should await further research. In the IMRT era, the risk of INN seems higher, which may be due to advanced techniques enabling target volumes to receive high-dose radiation. These INN patients experience foul nasal odor and persistent headache generally in the previously 2D/3D conventional therapy era [6]. Someone even suffers from internal carotid artery exposure or osteoradionecrosis, which increases the risk of death by 55.5–60.9% [7]. The benefits of treatments including endoscopic debridement and nasopharyngeal irrigation are also limited for severe-grade INN, as the previous study reported that only 28.6% of patients could be cured [8, 9]. Therefore, prevention is much important than management.

Quite a few studies have been reported risk factors to predict INN. The accumulated total dose of radiation, malnutrition, and infection are generally recognized as predictive factors [7, 10–12]. Yu developed a model including gender, pretreatment necrosis, accumulated total prescription dose, and recurrent tumor volume to predict INN in NPC patients with re-irradiation [13]. Li also established a nomogram to help clinicians distinguish the high-risk INN population, composed mainly of inflammatory and nutritional factors [11]. However, most previous studies included cases treated with two-dimensional (2D) radiotherapy and focused on the incidence of INN after re-irradiation. What is more, whether the clinical features of INN show differences from the 2D era and the predictive factors, especially recently combined treatment strategies, are of importance to the occurrence of INN remains unclear.

Our study analyzed INN in primary NPC patients treated with IMRT, aiming to define the clinical characteristics and the influence of treatment strategies on INN occurrence. It could help physicians better understand and prevent the development of INN in the IMRT era.

## Patients and methods

### Patient selection

Patients pathologically diagnosed with nasopharyngeal necrosis between January 2008 and December 2019 at our center were retrospectively reviewed. The inclusive criteria were as follows: (1) pathologically diagnosed nasopharyngeal cancer; (2) received radical IMRT for primary lesion; (3) diagnosis of INN was mainly based on clinical characteristics, endoscopic examination, and magnetic resonance imaging (MRI); 4) developed INN after primary irradiation without local recurrence; (5) full record of tumor treatment. It was required to distinguish INN from tumor necrosis-induced ulcers, which usually accompany tumor tissue.

### Treatment for primary tumor

Gross tumor volume (GTV) included primary gross disease, and clinical target volume (CTV) included the risk regions of microscopic disease. The high-risk regions of tumor invasion and nodal involvement were defined as CTV1. Low-risk nodal regions were defined as CTV2. Each volume's planning target volume (PTV) was determined with an additional 3-mm margin to generate PGTV, PTV1, PTV2. The simultaneous integrated boost technique was used, and the prescribed dose was 69.96 Gy in 33 fractions to PGTV for the T1–2 stage and 73.92 Gy for the T3–4 stage. The dose delivered to PTV1 was 60.06 Gy in 33 fractions, and to PTV2 was 50.96 Gy in 28 fractions. More than 110% of the prescription dose was not allowed to exist into or out of PTV. If a solid residual tumor exists after the completion of irradiation, a boost dose can be accessed with the IMRT technique.

Neoadjuvant (NC), concurrent (CC), or adjuvant chemotherapy (AC) adding to radiotherapy (RT) was given to patients with stage T3-4 or N1-3 disease. For stage II to IVa disease, CCRT as the backbone was recommended. NC or AC was alternatively added to patients with stage III–IVA disease, in which NC was preferred in high-risk patients for distant failures, such as the N3 stage or T4N2 stage. The NC regimen included two to three cycles of TP (docetaxel 75 mg/m2/day, day 1, cisplatin 25 mg/m2/day, days 1–3). CC consisted of 100 mg/m2 of cisplatin every three weeks for 2–3 cycles or 30 mg/m2 each week for 5–7 cycles. AC included two to four cycles of TP. To identify whether adding chemotherapy to CCRT contributes to INN occurrence, locally advanced NPC (stage T3–4N1–3) patients were divided into three treatment groups: NC + CCRT, CCRT + AC, CCRT.

Targeted therapy could be combined with NC, CC, or RT alone. Nimotuzumab was administered weekly as a 200 mg flat dose in 250 mL normal saline over 60 min. During radiotherapy, a total of 7 doses (1 per week) were administered.

### Evaluation and treatment for INN

During radiotherapy, patients underwent blood and biochemical testing, weight measurement (once a week), electronic nasopharyngoscopy and MRI (at middle and end of radiotherapy). After treatment, patients were required regular follow-up, conducted every 3 months during the first 2 years, 6 months during the next 3 years, and 1 year after 5 years. The events including INN, local–regional failure, distant metastasis and death were recorded.

The interval of getting INN was defined from the first day of initial radiotherapy to the first day of diagnosis. According to the depth of the necrosis on MRI imaging, nasopharyngeal necrosis was described as three stages: (1) early-stage: necrosis was confined to the mucosa; (2) middle stage: pathologic changes invading, but not exceeding the soft tissue; (3) severe stage: skull-based osteoradionecrosis occurs [8]. MRI examinations were diagnosed by two independent radiographers.

The treatment of INN was based on its severity. The patients with an early or middle stage INN were administered a conservative treatment and follow-up examination, including nutritional support, flushing with 0.9% saline and 1–2% hydrogen peroxide or chymotrypsin, and anti‑inflammation treatment at least for one month. For deep or sustained ulcers, repeated endoscopic debridement of the nasopharyngeal necrotic tissues was required, which was helpful to control infections, inhibit necrosis, and relieve symptoms of headache and foul odor. The patients with a severe stage INN or internal carotid artery exposure were treated with endoscopic debridement and anti‑inflammation treatment or endoscopic surgery.

### Statistical analysis

We developed a propensity score to balance essential variables for the comparative analyses between the INN and non-INN groups. According to previous studies, predictive factors including T stage, N stage, age, gender, and baseline hemoglobin were included in a logistic regression model to estimate the propensity score. A 1:4 matching with replacement and a caliper width equal to 0.2 of the standard deviation of logit of propensity score was performed. Then the patients without INN in our hospital from 2006 to 2017 were selected as the non-INN group. Baseline characteristics were compared within the two groups using the Chi-square test for categorical variables and the t-test for continuous variables. The Kaplan–Meier method was used to evaluate the overall survival (OS) and progression-free survival (PFS). Treatment strategies were subjected to univariate and multivariate logistic regression analysis to determine their associations with INN. The cut-off value regarding radiation dose was determined by using receiver operating characteristic (ROC) analysis. *p* < 0.05 was considered statistically significant. Statistical Package for Social Science software (SPSS, version 23.0) and R (version 4.0.4) were used.

## Results

### Basic patient characteristics

**Fig. 1:**
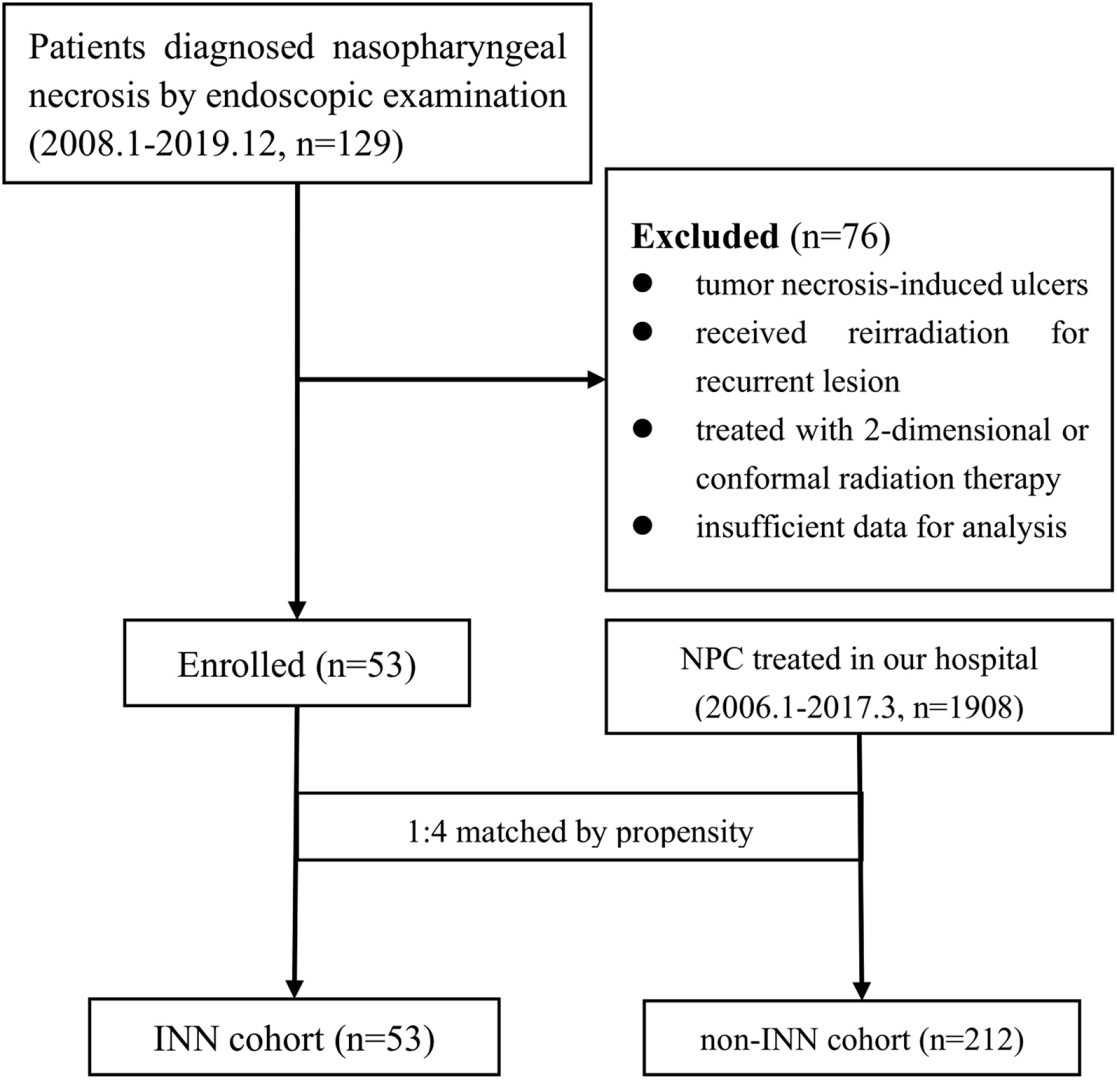
Flowchart of patient enrollment

Data from 2787 primary NPC patients treated with IMRT were reviewed. Finally, 53 (1.9%) patients who developed INN were collected (Fig. 1), with a median age of 55 (range from 17 to 78) years. The male gender (39, 73.6%) was more frequent. Before treatment, 17 (32.1%) patients had comorbidities, like anemia, hypertension or diabetes and 7 (13.2%) experienced necrosis caused by tumor. Overall, most patients (84.9%) were initially staged T3-4. The mean body mass index was 24.3 ± 3.8 (range from 16.0 to 33.7). The majority of patients (86.8%) presented weight loss, the average of which was 3.3 kg at the end of radiotherapy. Moreover, the body weight decreased by more than 5% in nearly half of the patients (26, 49.1%). The hemoglobin level decreased after radiation, and 17 (32.1%) patients showed mild anemia. The INN was predominantly distributed at the T3/4 stage (45, 84.9%). Of the INN population, 10 (18.9%) patients underwent targeted therapy, 37 (69.8%) underwent chemotherapy, including neoadjuvant chemotherapy for six patients, concurrent chemotherapy for 34 patients, and adjuvant chemotherapy patients. Six patients received VMAT, 15 received TOMO, and 32 received IMRT. Eight (15.1%) patients received a nasopharyngeal boost of 4–15 Gy due to partial response or stable disease after radiation. The boost dose was 4 Gy for 1 case, 8 Gy for 5 cases, and 15 Gy for 2 cases. The mean GTV dose was 74.78 Gy (median, 73.92 Gy; range, 69.96–89.45 Gy), with a median volume of 57.48 cm3 (range, 7.22–217.34 cm3).

### Clinical feature and prognosis of INN

Most cases (84.9%) occurred within two years after radiotherapy, of which the median time was 8 months, ranging from 0 to 147 months. 71.7% of cases had pseudomembrane during or at the end of radiotherapy. Headache (33, 62.2%) and foul odor (16, 30.2%) were the main symptoms, which in mild degree, while a few patients experienced intermittent epistaxis (4, 7.5%). According to the definition of INN staging, all patients were in early or middle stage INN and no one presented with skull-based osteoradionecrosis (serve stage). Three patients underwent surgical debridement and reconstruction, while others were given medical approaches and/or endoscopic debridement and irrigation of the nasal cavity. At the end of the follow-up, the median healing time was four months (1–16 months). In the patients with middle stage INN, four experienced nasal hemorrhage with internal carotid artery exposure; only one died of sudden nasopharyngeal massive bleeding. Five patients suffered repeated INN, and the median duration from initial INN occurrence was 15 months (9–58 months). All were with advanced T stage (one with T3, four with T4). Three patients had initial INN in the middle stage, and two of them suffered from massive bleeding, cured by endoscopic surgery.

**Fig. 2:**
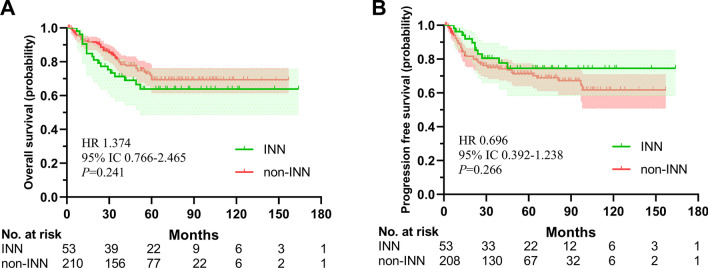
Kaplan–Meier curves of overall survival and progression-free survival of the INN group and non-INN group after propensity score matching

After propensity score matching, we were able to match all 212 non-INN patients to 53 INN patients (Fig. 1). T stage, N stage, age, gender, and baseline hemoglobin was well balanced with post-matching c-statistic. The clinical characteristics of patients in two groups were shown in Table 1. According to the K–M survival analysis, INN was not a prognostic risk factor, though the INN group had a lower estimated 5-year OS rate than the non-INN group (66% vs. 75.2%, *p* = 0.194). Similarly, the estimated rates of 5-year PFS (74.6% in the INN group, 71.3% in the non-INN group; *p* = 0.415) showed no statistical difference between the two groups (Fig. 2).

**Table 1:** Characteristics of NPC patients with INN

Characteristic	Original data set (n = 53)	Matched data set n = 212)	*p*
Gender			0.511
Male	39 (73.6)	165 (77.8)	
Female	14 (26.4)	47 (22.2)	
Age (y)			0.442
Median; Range	55; 17–78	55; 18–83	
Comorbidity			
Anemia	2 (3.8)	–	
Hypertension	10 (18.9)	–	
Diabetes	7 (13.2)	–	
Pathology			
Non-keratinizing undifferentiated carcinoma	34 (64.2)	122 (57.5)	
Non-keratinizing differentiated carcinoma	18 (34)	89 (42)	
Keratinizing carcinoma	1 (1.9)	1 (0.5)	
Necrosis before treatment			
Yes	7 (13.2)	–	
No	46 (86.8)	–	
HGB before treatment			0.139
Median; Range	143 (82–181)	142;91–182	
Pseudomembrane during radiation			
Yes	38 (71.7)	–	
No	15 (28.3)	–	
T staging			0.93
T1	3 (5.7)	16 (7.5)	
T2	5 (9.4)	17 (8.0)	
T3	18 (34)	77 (36.3)	
T4	27 (50.9)	102 (48.1)	
N staging			0.911
N0	9 (17.0)	35 (16.5)	
N1	17 (32.1)	73 (34.4)	
N2	20 (37.7)	83 (39.2)	
N3	7 (13.2)	21 (9.9)	
Stage			
I	0	3 (1.4)	
II	6 (11.3)	7 (3.3)	
III	16 (30.2)	82 (38.7)	
IV	31 (58.5)	120 (56.6)	
Chemotherapy			
No	16 (30.2)	68 (32.1)	
Neoadjuvant	6 (11.3)	30 (14.2)	
Concurrent	34 (64.2)	106 (50.0)	
Adjuvant	4 (7.5)	8 (3.8)	
Target therapy			
Yes	10 (18.9)	66 (31.1)	
No	43 (81.1)	146 (68.9)	
Local boost			
Yes	8 (15.1)	5 (2.4)	
No	45 (84.9)	207 (97.6)	
INN stage			
Early	32 (60.4)	–	
Middle	21 (39.6)	–	
Serve	0	–	

*NPC* nasopharyngeal carcinoma, *INN* Irradiation-induced nasopharyngeal necrosis, *HGB* hemoglobin

### Association between treatment strategies and INN

Using univariate and multivariate logistic regression analysis, we determined that adding neoadjuvant, concurrent, adjuvant chemotherapy or molecular targeted therapy had no significant impact on INN occurrence. Neither did various treatment strategies of chemoradiotherapy locally advanced NPC patients (shown in Table 2). However, patients receiving boost doses were more accessible to develop INN than those who did not (61.5% (8/13) vs. 17.9% (45/252); OR 7.360, 95% CI 2.301–23.547; *p* = 0.001). When boost dose ≥ 8 Gy, more patients (77.8% vs. 25%, *p* = 0.217) had INN. Though accumulated dose was an independent predictor of INN, the optimal threshold for it was failed to define using ROC curves (AUC = 0.521) from the derivation data of 265 patients.

**Table 2:** Univariate and multivariate analysis of relationship between treatment strategies and INN occurrence

Variables	Univariate		Multivariate	
	OR (95% IC)	*p *value	OR (95% IC)	*p *value
No chemotherapy	1.092 (0.568–2.099)	0.792	3.035 (0.398–23.162)	0.284
AC	2.082 (0.602–7.194)	0.247	2.099 (0.530–8.315)	0.291
NC	0.632 (0.233–1.716)	0.368	0.554 (0.171–1.790)	0.324
CC	0.940 (0.501–1.763)	0.847	0.387 (0.057–2.605)	0.329
Target therapy	0.647 (0.320–1.312)	0.227	0.536 (0.252–1.141)	0.106
Local boost	7.360 (2.301–23.547)	0.001	7.768 (2.340–25.785)	0.001
Chemotherapy strategies (NC + CCRT, CCRT + AC, CCRT)	–	0.475	–	–

*AC* adjuvant chemotherapy, *NC* neoadjuvant chemotherapy, *CC* Concurrent chemotherapy, *RT* radiotherapy

## Discussion

INN as a severe complication can present during or after radiotherapy on NPC patients. However, little is known about the outcomes for its extremely low incidence in the IMRT era. The clinical characteristics and prognosis of INN are different from those in the 2D era. In this study, most cases had pseudomembrane during or at the end of radiotherapy. Moreover, the severity of INN was lessened than previous studies, which seemed to have little effect on patients’ survival under the treatment of anti-inflammation, nasal flushing and nutritional support. Our data also determined that combined with chemotherapy or target treatment was not associated with developing INN after initial radiation. In contrast, the accumulative radiation dose was the only independent risk factor.

The crude incidence rate of INN was 1–2.9% among the NPC population [11, 12, 14], and 3.3% in patients treated with IMRT while 2.3% with 2D radiotherapy [11]. Compared to 2D radiation therapy, IMRT enables target volumes to receive higher doses, which may increase the incidence of INN. Notably, these studies enrolled patients with local recurrence receiving re-irradiation, which contributed to INN occurrence. As reported, 15.2–31.5% of locally recurrent patients would experience INN following re-irradiation with IMRT [13, 24, 25]. Instead, INN is rare for the primary NPC; only 1.9% of patients treated at our center developed INN after first irradiation. Several literatures tried to explain the mechanism of INN. A theoretical deduction accepted wildly is that chronic repair cannot compensate for tissue breakdown. Infection, hypoxia, hypervascularity, and nutritional deficiency are reported as essential roles in the process of INN [7, 11]. Our study showed that most patients (38/53, 71.7%) had pseudomembrane at the middle or the end of radiotherapy. It can provide conditions easy to infection, which impairs the normal nasopharynx mucosa, increases hypoxia status and aggravates the damage. Therefore, pseudomembrane may predict the occurrence of INN.

As previously reported, most INN patients would suffer different degrees of symptoms like headache, foul odor and recurrent bleeding [9]. Our data showed that patients mainly manifested mild headaches and foul odor, few of whom needed powerful pain relievers. To analyze the severity, INN was ranked into three stages according to MRI characteristics. Previous studies indicated that significant patients suffered INN in the middle or severe stage. Deep parapharyngeal ulcer could invade the internal carotid artery, which may cause fatal bleeding and death. 26.9–45% of patients with INN got the internal carotid artery involved, resulting in severe adverse event [7, 15]. In the devastating late complications, the incidence rate of osteoradionecrosis was even up to 10.1% [16]. Moreover, internal carotid artery exposure and osteoradionecrosis have been identified as prognostic factors impacting the quality of life and endangering life. However, INN also showed less severity in our study than in previous trials. No one developed osteoradionecrosis, and 62.2% of patients were in the early stage, which only calls for conservative or medical treatment. The main reason may be that published reports enrolled many cases receiving re-irradiation at the primary site. Of these patients, 44% had grade ≥ 3 INN [17]. In patients with middle-stage INN, four had sudden massive bleeding, and one died from a blood vessel burst. Traditional repeated endoscopic debridement is generally applied for patients with carotid artery exposure and rupture, but only 13.4–28.6% of patients can be cured [18]. Even novel surgical management achieved limited clinical efficacy [9, 18, 19]. However, our study revealed that INN was not an independent prognostic factor for OS and PFS, which indicated that INN had limited influence on patients’ survival after the primary IMRT. While there was a trend of a higher rate of PFS in the INN group, which may be due to higher dose to local region contributing to local control, then a trend of a lower rate of OS was also shown, which may be ascribed to severe INN. The low severity levels of INN and better treatment approaches nowadays may have a minor effect on the survival outcomes.

Intensive treatment strategies, such as combining neoadjuvant, adjuvant chemotherapy or molecular targeted therapy with concurrent chemoradiation, may increase the local tissue damage, which aggravates irradiation-induced injuries and hinders the healing of the ulcer. At worse, the survival benefit of these strategies is uncertain for locally advanced NPC [20–22]. Therefore, we aimed to investigate whether the intensive treatment strategies would increase the risk of INN occurrence. A propensity-score matching methodology was used to balance other potential risk factors, such as T stage, gender, age, pathological type, biomarkers of nutritional status (hemoglobin, albumin, body mass index), and inflammation status (C-reactive protein, necrosis before re-irradiation), which were reported by published researches [11–13, 17]. As a result, our study noted that adding chemotherapy or targeted therapy did not increase the risk of developing INN, as in line with other studies [11, 23]. However, the response to neoadjuvant chemotherapy may be a predictor. Yan et al. showed that patients with stable disease response to neoadjuvant chemotherapy were more prone to suffer INN than those with partial response [23]. The poor response revealed the blood and oxygen supply deficiency in primary tumor tissue, less radiosensitivity, and lower recovery capability.

Furthermore, the accumulated prescription dose to the GTV plays a vital role in the INN occurrence. Hua et al. observed that a total dose over 120 Gy of 2 courses of radiation was significantly associated with INN. Similarly, Yu et al. reported that a third of patients with INN received an accumulated dose over 141.5 Gy [13]. However, a lower rate of INN occurs in patients with initial radiation, and whether the radiation dose is a risk factor needs to be determined. Li et al. indicated that the D3cc was an independent predictor for INN in primary NPC patients, which should be limited under 73.67 Gy [26]. On the contrary, Fei et al. reported that dose-volume (tumor volume and ratio of tumor volume exposed to 74 Gy to GTV) did not affect the incidence of INN, neither did boost doses, which seems incompatible with clinical experience [12]. Given the small sample (only nine patients developed INN in this study), the result should be hardly definitive. In our series, both univariate and multivariate analysis showed that boost dose was related to INN occurrence, following other studies. However, the optimal cut-off point of accumulated dose could not be identified by ROC analysis for the poor prediction efficiency (AUC = 0.521). Due to the occurrence of INN, it was associated with many other factors, only one achieving insufficient prognostic capacity.

This study also has some limitations. Firstly, we enrolled a small sample of patients due to the low incidence of INN. Small group population will reduce prediction capability, and further studies should confirm the forecasting performance. Then the follow-up was irregular which contributed to the insufficient clinical information of some patients. The various indicators of nutrition and infection status, including the dynamic change, could make the analysis more complicated. It is hard to contain all the potential risk factors. What is more, as a single-center retrospective study, selection bias cannot be avoided.

## Conclusion

NPC patients initially treated with IMRT suffer INN less severely than those with reirradiation or 2D/3D techniques. Combining chemotherapy or targeted therapy with radiotherapy does not increase the risk of developing INN. However, the accumulated dose of radiation is associated with it. Further investigations are required to identify independent predictors of INN for primary NPC patients in the IMRT era.

## Data Availability

The datasets used and/or analyzed during the current study available from the corresponding author on reasonable request.

## Abbreviations

AC: Adjuvant chemotherapy
CC: Concurrent chemotherapy
CTV: Clinical target volume
GTV: Gross tumor volume
IMRT: Intensity-modulated radiotherapy
INN: Induced nasopharyngeal necrosis
MRI: Magnetic resonance imaging
NPC: Nasopharyngeal carcinoma
NC: Neoadjuvant chemotherapy
OS: Overall survival
PFS: Progression-free survival
PTV: Planning target volume
RT: Radiotherapy
ROC: Receiver operating characteristic
TOMO: Tomotherapy
